# Supplementary material for: Thin lamellar films with enhanced mechanical properties for durable radiative cooling
Source: Nat Commun. 2023 Oct 2;14:6129. doi: 10.1038/s41467-023-41797-3 (PMC10545832; doi:10.1038/s41467-023-41797-3)
Supplement: Supplementary file 1 — Supplementary Information [file 41467_2023_41797_MOESM1_ESM.pdf]

Supplementary Information

Thin lamellar films with enhanced mechanical properties for durable radiative cooling

Lianhu Xiong[1], Yun Wei[1], Chuanliang Chen[1], Xin Chen[1], Qiang Fu[1], Hua Deng[1]**

[1]College of Polymer Science and Engineering, State Key Laboratory of Polymer Materials Engineering, Sichuan University, Chengdu, 610065, China

*Corresponding authors: qiangfu@scu.edu.cn (Q. Fu), huadeng@scu.edu.cn (H. Deng)

This PDF file includes:

Supplementary Notes S1 to S4

Supplementary Figs. 1 to 28

Supplementary Table 1

Supplementary References

Other Supplementary Materials for this manuscript include the following:

Supplementary Movies 1 to 3 (.mov)

Supplementary Notes

Note S1: Finite-Difference Time-Domain Simulations of Scattering Behaviors

To evaluate the light scattering behaviors of dielectric scatterers, Finite-Difference Time-Domain (FDTD) simulations were carried out using FDTD Solutions 8.19 software by Lumerical Co. Ltd. Three-dimensional geometric models were established according to the real situation, for example, core-shell cube for Mica@TiO_2 and sphere for TiO_2, and corresponding complex refractive index (k) data was obtained from the public database. To investigate the influence of scatterer size, we further regulated the thickness and diameter of Mica or TiO_2 layer based on the above models. Across the UV-VIS-NIR band (0.3-2.5 µm), scattering cross-section (C_{sca}), near-field electric field distribution and far-field scattering phase function of different scatterers were simulated using total-field scattered-field (TFSF) source and perfectly matched layer (PML) boundary condition. Then, the scattering efficiency (Q_{sca}) is the normalization of C_{sca} as[1]:

$$Q_{sca} = \frac{C_{sca}}{A} \quad \text{(S1)}$$

where A is the geometrical cross-sectional area of scatterers. The forward or backward scattering direction in the far-field scattering phase function is defined on the basis of subjectively specified datum-plane, as shown in Supplementary Fig. 16, for example, on the XZ section, with regard to the incident direction of backward z-axis (90°), 0-180° and 180-360° represent the backward scattering and forward scattering, respectively.

Note S2: Full-Field Electromagnetic Simulations by Finite-Element Method

Numerical full-field electromagnetic simulations were performed based on the finite-element method (FEM) using commercial software package COMSOL Multiphysics 5.6 with "Electromagnetic Waves, Frequency Domain" physics interface of the wave optics module. Porous structures are built by randomly introducing square air voids with different pore size distributions in two-dimensional cross-section, thus simulating layered AMTA with interlamellar micropores as shown in Supplementary Fig. 19d-f. Although, by assumption, space extends to infinity in horizontal direction, it is sufficient to model a small sectional unit cell. Owing to the periodicity of the solution along the interface, a Floquet-periodic boundary condition is applied on the left and right unit-cell boundaries, emplaced with PML on the top and bottom sides. Subsequently set up the physics based on the direction of propagation and the E-field polarization. Periodic port conditions are excited through free space both for specifying the incident plane wave of 1 W/m (P_{in}) and for authorizing the resulting solution leave the model without any nonphysical reflections. Afterward, "Electromagnetic Waves, Frequency Domain" equation can be solved by FEM using non-homogeneous Helmholtz equation:

$$\nabla \times \mu_r^{-1}(\nabla \times E) - k_0^2 \varepsilon E = 0 \tag{S2}$$

where μ_r is the relative permeability, E is the electric field, k_0 is the propagation wave vector and $\varepsilon = (n - ik)^2$, n and k are the real and imaginary parts of the complex refractive index, respectively. Finally, all the reflectivity (R) and transmissivity (T) for incident plane wave can be calculated using reflected and transmitted power divided by incident power:

$$R = \frac{P_{ref}}{P_{in}} \tag{S3}$$

$$T = \frac{P_{tra}}{P_{in}} \tag{S4}$$

where P_{ref} and P_{tra} are the reflected and transmitted power integrated from the top and bottom ports, respectively. Besides, note that for the law of electromagnetic wave transfer in different porous models, the electric field norm distribution is a suitable perspective, which can be obtained simultaneously in the parametric sweep for each wavelength (0.3-2.5 μm).

Note S3: Theoretically Calculated Radiative Cooling Power

To better understand the radiative cooling performance of AMTA, the net cooling power (P_{net}) for subambient and above-ambient conditions is systematically calculated by:

$$P_{net}(T_{rad}) = P_{rad}(T_{rad}) - P_{atm}(T_{amb}) - P_{sun} - P_{cond+conv} \qquad \text{(S5)}$$

where P_{rad} is the radiative power emitted from AMTA, calculated by:

$$P_{rad}(T_{rad}) = \int d\Omega \cos\theta \int I_{BB}(T_{rad}, \lambda)\, \varepsilon(\lambda, \theta) d\lambda \qquad \text{(S6)}$$

in which, $\int d\Omega = 2\pi \int_0^{\pi/2} \sin\theta d\theta$ is the hemispherical angular integral, $\varepsilon(\lambda, \theta)$ is the measured spectral emissivity of AMTA as a function of wavelength (λ) and incident angle (θ), $I_{BB}(T_{rad}, \lambda) = \frac{2hc^2}{\lambda^5} \frac{1}{e^{hc/(\lambda k_b T_{rad})} - 1}$ is the spectral irradiance emitted by a blackbody, where T_{rad} is the temperature of radiative coolers, c is the light speed, h is the Plank constant and k_b is the Boltzmann constant. P_{atm} is the absorbed atmospheric radiation, calculated by:

$$P_{atm}(T_{amb}) = \int d\Omega \cos\theta \int I_{BB}(T_{amb}, \lambda)\, \varepsilon(\lambda, \theta)\, \varepsilon_{atm}(\lambda, \theta) d\lambda \qquad \text{(S7)}$$

in which, T_{amb} is the temperature of ambient air, $\varepsilon_{atm}(\lambda, \theta) = 1 - [\tau_{atm}(\lambda, \theta)]^{1/\cos\theta}$ is the spectral directional emissivity of atmosphere, where $\tau_{atm}(\lambda, \theta)$ represents the atmospheric transmissivity at vertical direction. P_{sun} is the absorbed solar irradiance, calculated by:

$$P_{sun} = \int \varepsilon(\lambda, \theta_{sun})\, I_{AM1.5}(\lambda) d\lambda \qquad \text{(S8)}$$

in which, θ_{sun} is the incident angle of the sunlight, $I_{AM1.5}$ is the direct normal spectral solar irradiance ASTM G173 under air-mass 1.5. $P_{cond+conv}$ is the non-radiative power losses, calculated by:

$$P_{cond+conv} = q(T_{amb} - T_{rad}) \qquad \text{(S9)}$$

in which, $q = q_{cond} + q_{conv}$ is the non-radiative heat coefficient composed of the thermal conduction and convection.

Note S4: Microscale Finite-Element Analysis of Mechanical Response

A two-dimensional (2D) nonlinear finite-element model was conducted to investigate the uniaxial tensile mechanical response of porous lamellar structures at the microscale using the commercial software ABAQUS/CAE (v2016; Dassault Systemes Simulia, Johnston, RI, USA). For the simplified 2D plane-strain conditions, all mesoscopic units were assumed to follow a typical constitutive relation according to the literature[2,3] and experimental data. In the simulation, two porous lamellar microstructures with and without a single edge notch were established, as shown in Supplementary Fig. 12c,d. Specifically, the hard Mica@TiO_2 platelets were arranged together with an ideal distribution, forming a highly ordered lamellar microstructure, while the regular pores were simultaneously introduced into spacing layers, as observed in experiments. The dendritic ANFs adhesives between adjacent platelets were modeled as the zero-thickness interfaces with normal and tangential interfacial stiffness using the cohesive zone modeling (CZM) approach, which is usually employed to study the adhesion or fracture in different materials[4,5]. The constitutive relation of CZM is described by the bilinear traction-separation law in Supplementary Fig. 12a, where T, δ_c, δ_f, K and G_c represent interfacial strength, critical separation, failure separation, interfacial stiffness and critical fracture energy release rate, respectively.

Based on the constitutive equation, the hybrid fracture mode during mechanical damage can be determined by the following secondary nominal stress and strain criteria[6]:

$$\left\{\frac{\langle t_n\rangle}{t_n^0}\right\}^2+\left\{\frac{\langle t_s\rangle}{t_s^0}\right\}^2+\left\{\frac{\langle t_t\rangle}{t_t^0}\right\}^2=1 \tag{S10}$$

$$\left\{\frac{\langle \varepsilon_n\rangle}{\varepsilon_n^0}\right\}^2+\left\{\frac{\langle \varepsilon_s\rangle}{\varepsilon_s^0}\right\}^2+\left\{\frac{\langle \varepsilon_t\rangle}{\varepsilon_t^0}\right\}^2=1 \tag{S11}$$

where t_n, t_s and t_t represent the cohesive strength in the normal (Z) and tangential (X, Y) directions, respectively. Naturally, ε_n, ε_s and ε_t are the nominal strains corresponding to the stress. In this way, the models qualitatively characterized the primary deformation, damage evolution and failure fracture of porous lamellar microstructures within AMTA under uniaxial tension, which provided insights into the strengthening and toughening mechanisms of such hierarchical structures.

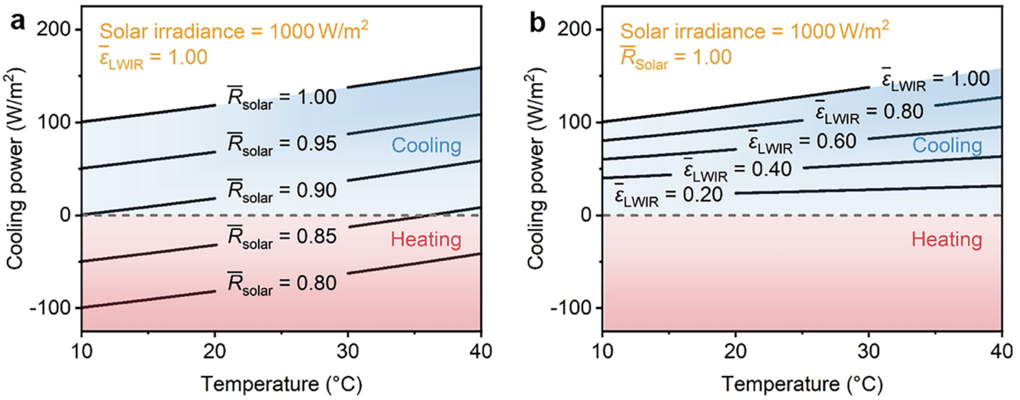

Supplementary Fig. 1. Cooling power as a function of ambient temperature for various $\overline{R}_{\text{solar}}$ at $\overline{\varepsilon}_{\text{LWIR}} = 100\%$ (**a**) and various $\overline{\varepsilon}_{\text{LWIR}}$ at $\overline{R}_{\text{solar}} = 100\%$ (**b**), respectively. Theoretical calculation demonstrates that emitting power is largely offset by the absorbed solar irradiance when $\overline{R}_{\text{solar}} < 90\%$ even if $\overline{\varepsilon}_{\text{LWIR}}$ reaches the theoretical limit, resulting subambient radiative cooling is difficult to achieve. On the contrary, if $\overline{R}_{\text{solar}}$ equals 100%, cooling mode is easier to access regardless of $\overline{\varepsilon}_{\text{LWIR}}$. Although the above discussion is based on the ideal materials without considering thermal conduction and convection, it also illustrates the necessity of high solar reflectivity in outdoor passive daytime radiative cooling (PDRC).

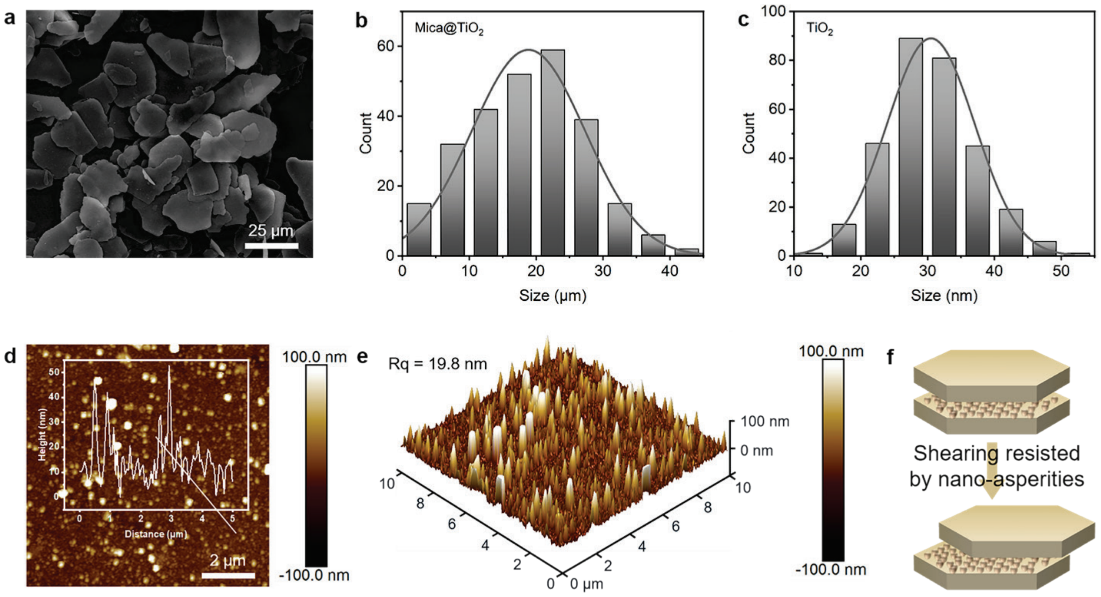

Supplementary Fig. 2. Characterization of Mica microplatelets with uniformly distributed TiO_2 nanograins (Mica@TiO_2). **a**) FESEM image of exfoliated Mica@TiO_2 microplatelets. **b,c**) Statistical diameter distributions of Mica@TiO_2 microplatelets (**b**) and TiO_2 nanograins (**c**), showing number-weighted mean sizes of ~20 μm for Mica@TiO_2 and ~30 nm for TiO_2. **d,e**) 2D (**d**, inset is a height image of the white straight line) and 3D (**e**) AFM images of Mica@TiO_2 surface, showing uneven vertical size ranging from 5 nm to 50 nm with root-mean-square roughness (Rq) of 19.8 nm. **f**) Extrinsic toughening through inelastic shearing resisted by rough nano-asperities in AMTA.

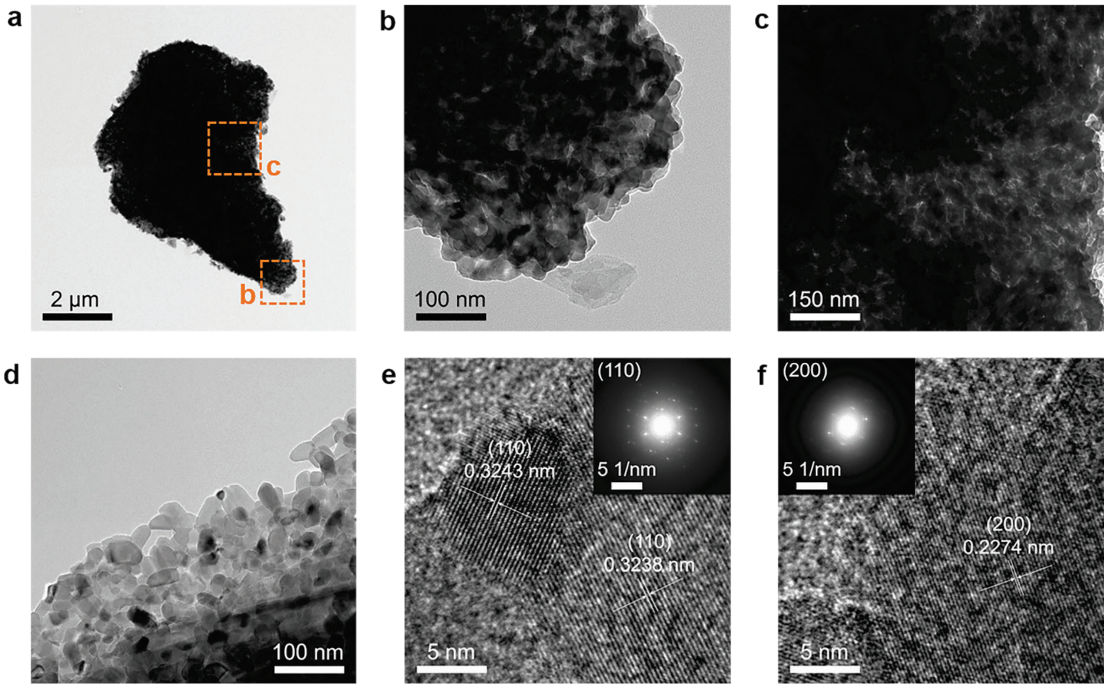

Supplementary Fig. 3. Characterization of the distribution and crystalline phase of TiO_2 on Mica surface. a) TEM image of the Mica@TiO_2, indicating that the mica platelet is completely coated with TiO_2 nanograins, which in turn forms a multi-interfaces core-shell structure. **b-d**) Corresponding magnified TEM images of TiO_2 nanograins on Mica surface, directly demonstrating their uniform and dense distribution, while high-magnification images at the Mica edges also prove that the thickness of the TiO_2 layer is about less than ~100 nm. **e,f**) High-resolution TEM images (HRTEM) and corresponding fast Fourier transform (inset images) of TiO_2 nanograins. Two types of diffraction patterns and lattice fringes are consistent with the (110) and (200) planes of the Rutile TiO_2[7], which is an ideal inorganic scatterer with higher refractive index (k ~2.7).

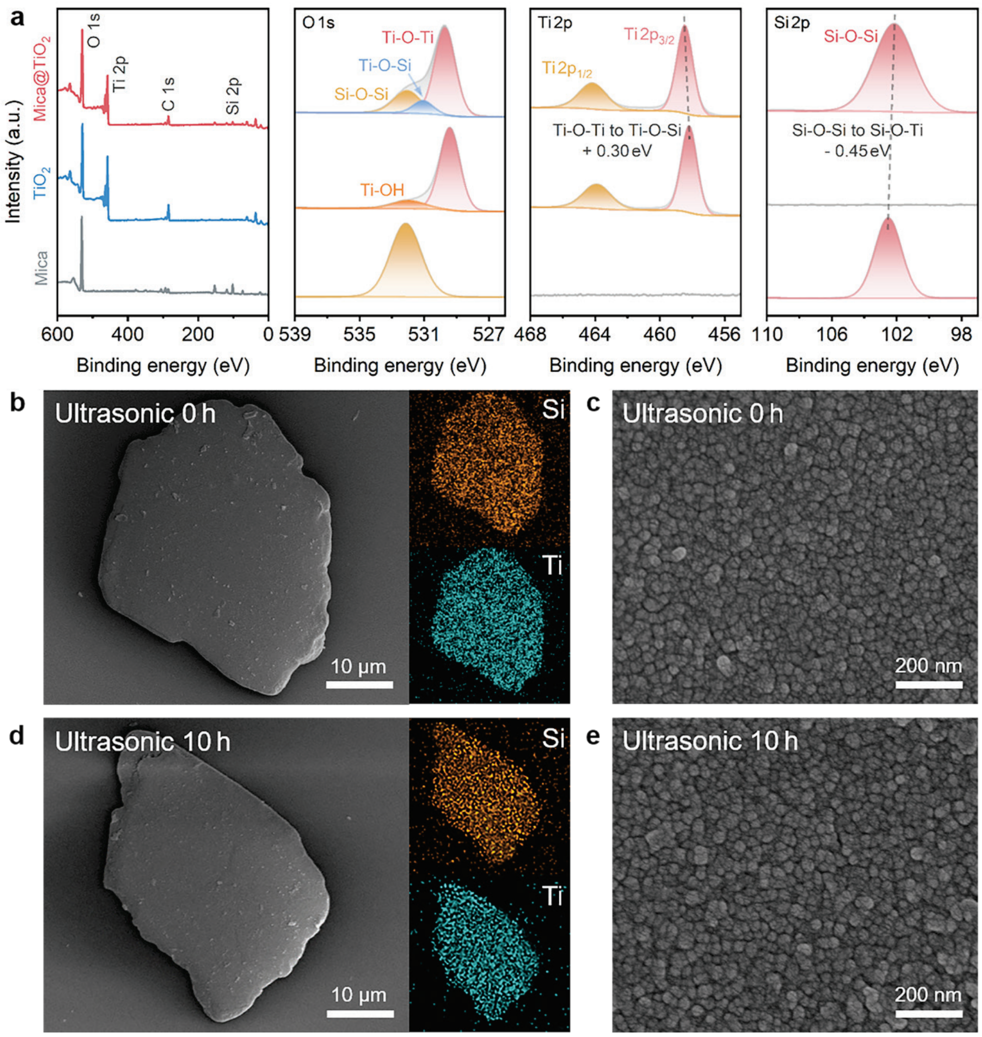

Supplementary Fig. 4. Stability of the combination of Mica and TiO_2. a) XPS full spectra and O1*s*, Ti2*p*, Si2*p* fine spectra of Mica, TiO_2 and Mica@TiO_2. In the O1*s* spectrum of Mica@TiO_2, a new Ti-O-Si peak (531.1 eV) can be observed, while the peak shift of Ti2$p_{3/2}$ (+0.30 eV) and Si-O-Si (-0.45 eV) is also captured in the Ti2*p* and Si2*p* spectra, respectively, which indicates that the Mica and its surface TiO_2 nanograins are not only physical binding, but a new type of chemical bonding (Ti-O-Si) is formed at the interface fusion, so that the two stable combination. **b-e**) FESEM images of Mica@TiO_2 (**b,d**) and TiO_2 nanograins (**c,e**) before and after long-term (10 h, 600 W) ultrasonic testing, showing that the TiO_2 on Mica surface did not undergo significant loosening or shedding under high-power ultrasonic damage, demonstrating the excellent bonding stability of both.

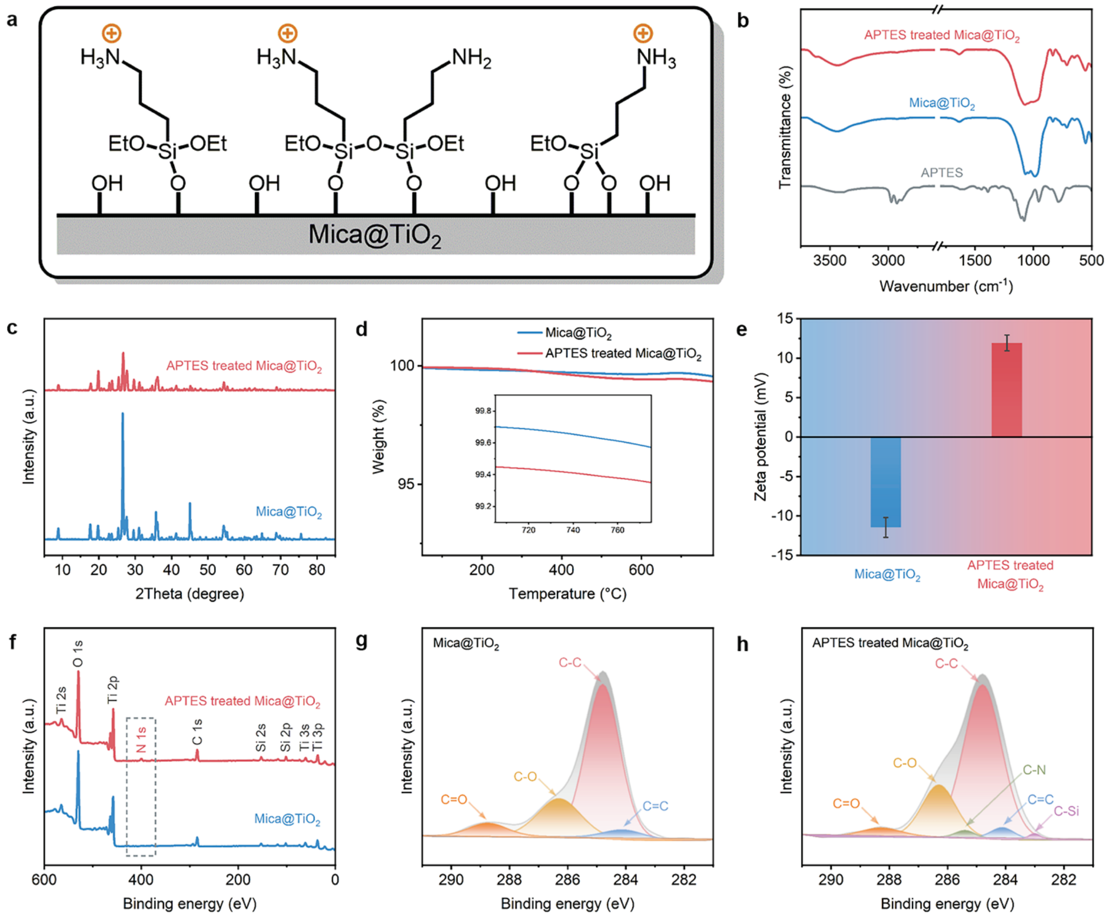

Supplementary Fig. 5. Characterization of Mica@TiO₂ modified by APTES. a) Mechanism diagram of Mica@TiO₂ modified by APTES, showing suspended hydroxyl groups on Mica@TiO₂ can undergo various bridging reactions with the silane groups of APTES. **b-d)** FT-IR (**b**), XRD (**c**) and TGA (**d**, inset is an enlarged view at 700 to 800 ℃) of Mica@TiO₂ before and after APTES modification, indicating silane coupling agents effectively worked. **e)** Zeta potential with absolute value of 12 mV had completely reversed after APTES treated, thus enhancing the electrostatic interaction with ANFs. **f-g)** XPS full-spectra (**f**) and C1*s* fine-spectra (**g,h**) of Mica@TiO₂ before and after APTES modification, indirectly explaining the coupling mechanism, namely the formation of new chemical bonds such as C-N and C-Si.

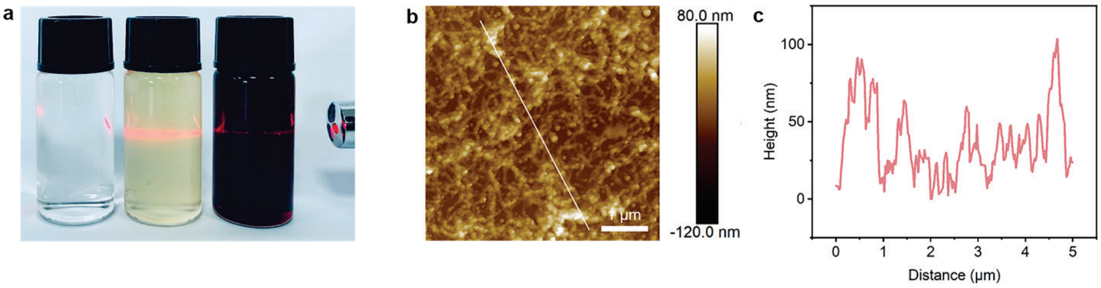

Supplementary Fig. 6. Characterization of hyperbranched ANFs. a) Tyndall phenomenon of ANFs dendritic colloid suspension (middle) processed by "Solvent exchange-Turbulent shear". The left and right are respectively water and ANFs/DMSO solution. **b**) AFM image of hyperbranched ANFs network with fibrillar joints, displaying highly interconnected joints with abnormal thickness, which is consistent with the OM and FESEM results (Fig. 1d,e). **c**) The height image of the white straight line in (**b**), showing the thickness at fibrillar joints can reach 100 nm. Therefore, the formation of ANFs network with high-density fibrillar joints directly improves the processing stability and final mechanical performance of the composites.

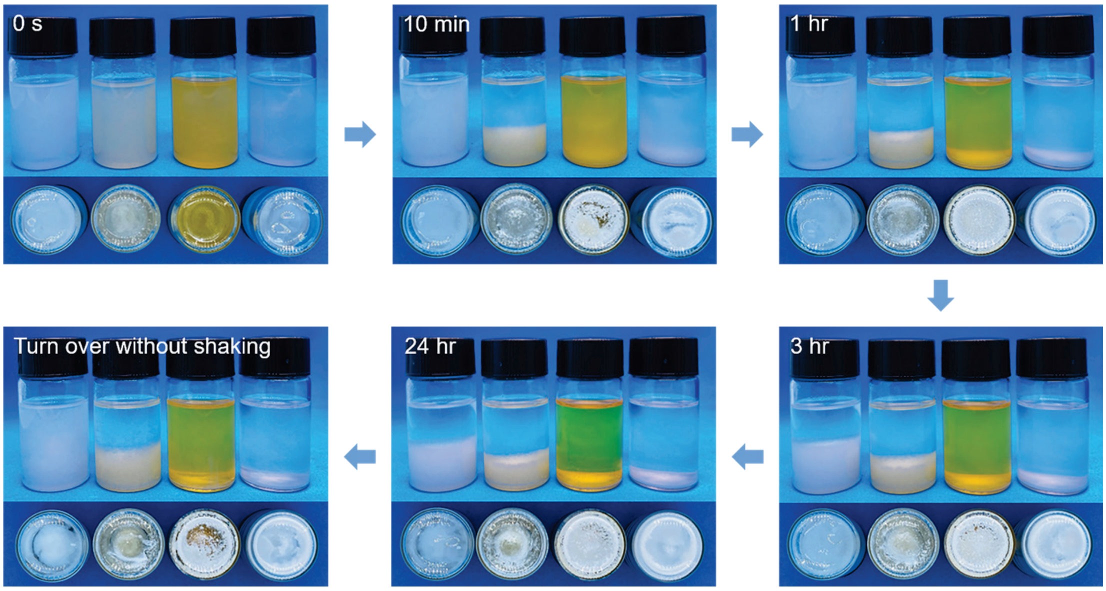

Supplementary Fig. 7. Stability of the ANFs/Mica@TiO_2 suspensions. Front and bottom photographs were taken at different moments after the suspensions were prepared. From left to right, four samples in photographs are respectively (1) ANFs/DMSO/Mica@TiO_2/IPA processed by "Solvent exchange-Turbulent shear", (2) ANFs/DMSO/Mica@TiO_2/H_2O processed by "Solvent exchange-Stirring" (conventional methods), (3) ANFs/DMSO/Mica@TiO_2 without solvent exchange and (4) Mica@TiO_2/DMSO. All suspensions contain the same Mica@TiO_2 loading, i.e. 90 wt%. It can be observed that the suspension processed by our method could be stably dispersed for several hours without any filler sedimentation, indicating the effect of hyperbranched ANFs network with fibrillar joints on trapping Mica@TiO_2 within the suspension. Furthermore, although the ANFs network would also deposit with Mica@TiO_2 to a certain extent after a long time, but it could easily return to the original state only by gently turning it over, which is far superior to other conventional processing methods, providing an industrial prospect for the subsequent preparation of film or slurry.

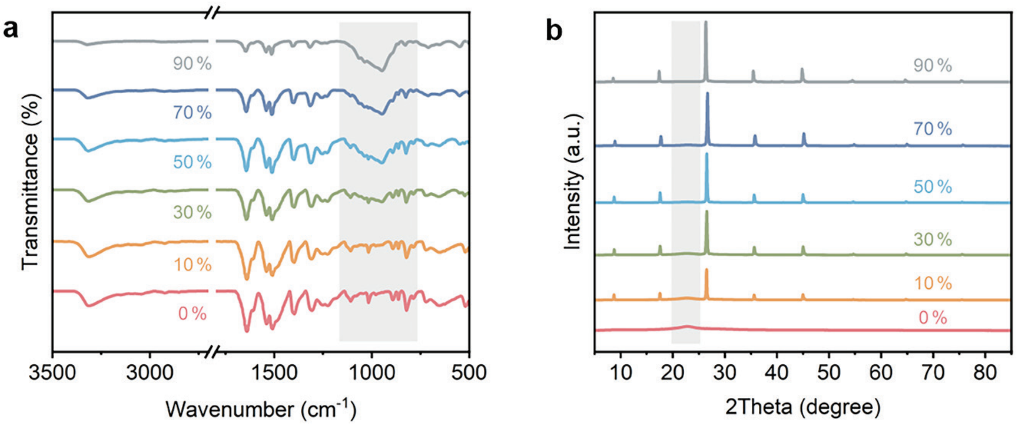

Supplementary Fig. 8. FT-IR and XRD spectra of ANF/Mica@TiO_2 composites. a) FT-IR spectra of ANFs/Mica@TiO_2 composites. With the increase of Mica@TiO_2 loading, the infrared vibration of N-H (~3300 cm^{-1}) and C=O (~1650 cm^{-1}) decreases, while the infrared absorption of Si-C, Si-O and Ti-O (~950 cm^{-1}) increases, which significantly enhances the emissivity of the PDRC materials in the atmospheric window (8-13 µm, gray area). **b**) XRD spectra of ANFs/Mica@TiO_2 composites. Typical peaks of lamellar composites with different Mica@TiO_2 loading can match those of pure ANFs and Mica@TiO_2. Moreover, the observed diffraction peaks of ANFs (gray area) are similar to those of the crystalline phases in AMFs[8], demonstrating the reservation of orientation structures in nanoscale domains.

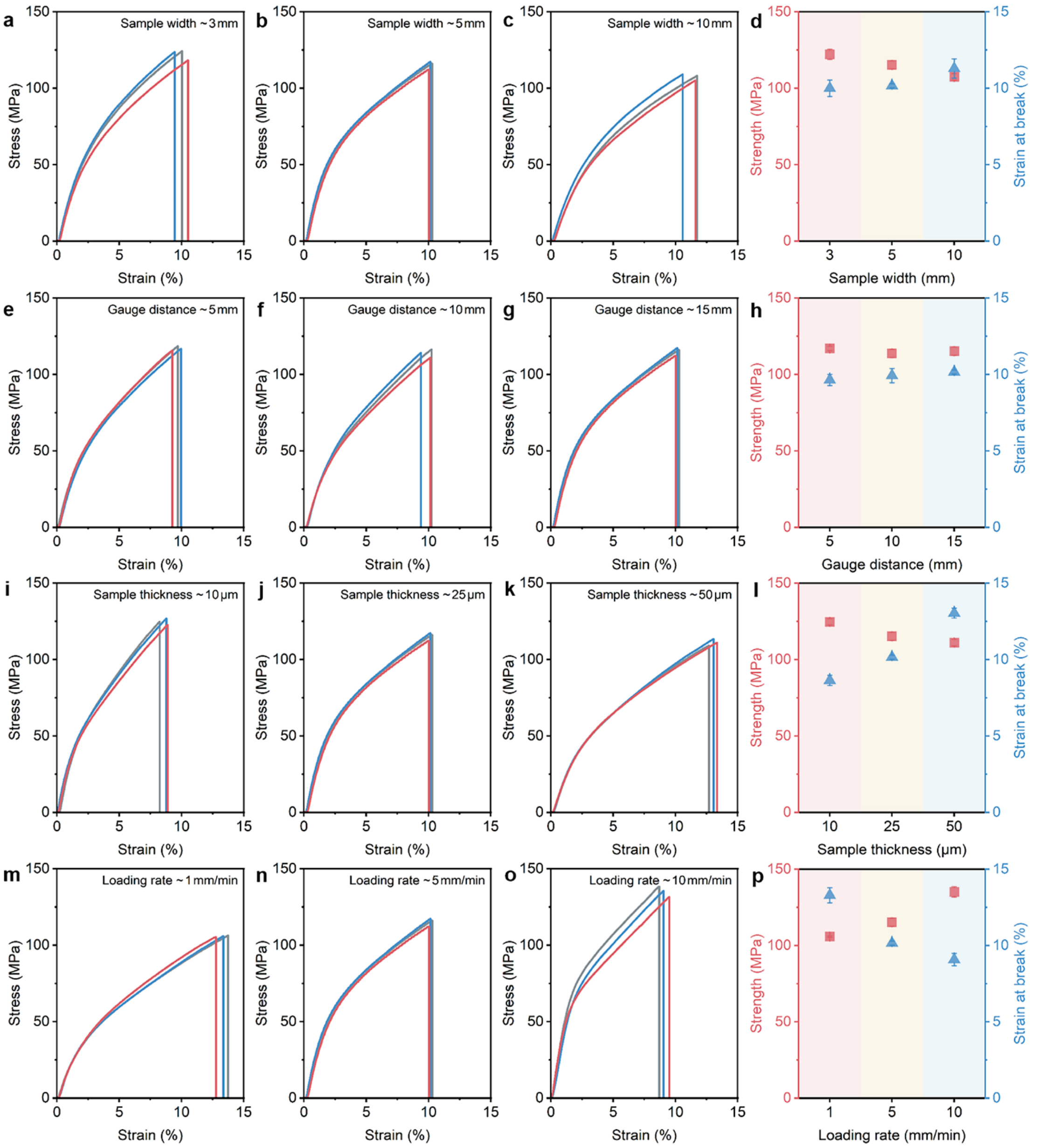

Supplementary Fig. 9. Tensile stress-strain curves of AMTA under different mechanical testing conditions. a-d) Sample width (3, 5, 10 mm). **e-h**) Gauge distance (5, 10, 15 mm). **i-l**) Sample thickness (10, 25, 50 μm). **m-p**) Loading rate (1, 5, 10 mm/min). Based on the above test results and literature standards[4,9,10] (ASTM D882, etc.), sample width ~5 mm, gauge distance ~15 mm, sample thickness ~25 μm and loading rate ~5 mm/min were selected as the final testing conditions, which can reflect the mechanical properties of AMTA more accurately and comprehensively.

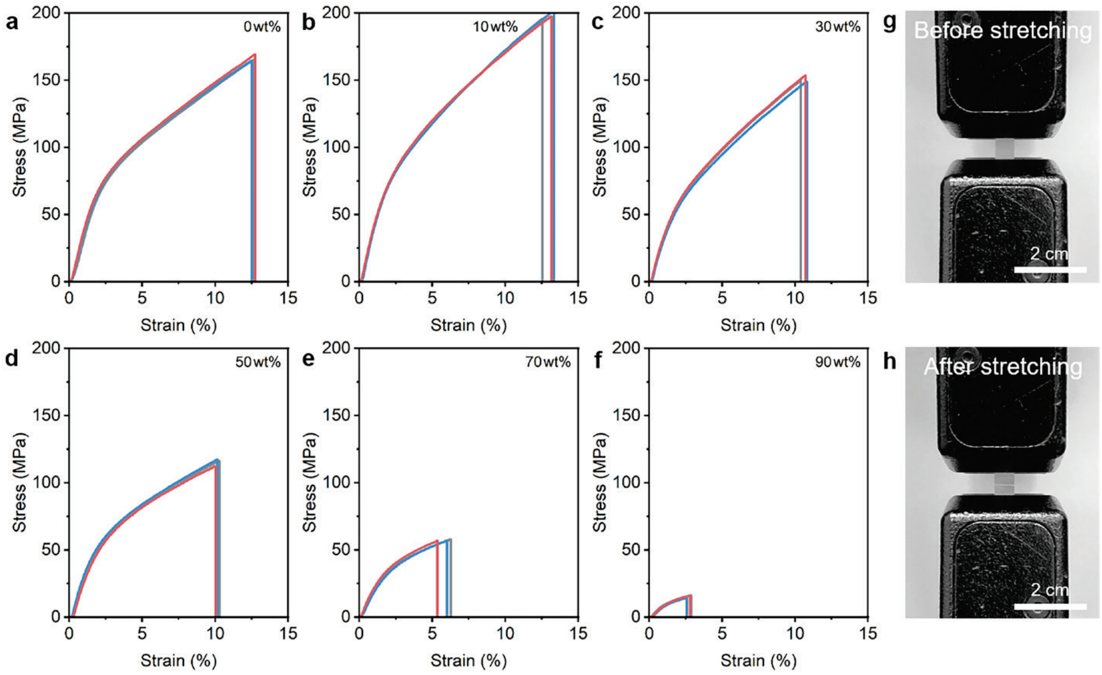

Supplementary Fig. 10. Tensile stress-strain curves of ANFs/Mica@TiO_2 composites. a-f) Tensile stress-strain curves of ANFs/Mica@TiO_2 composites with different scatterer loading (0, 10, 30, 50, 70, 90 wt%). **g,h**) Photographs of AMTA before (**g**) and after (**h**) stretching under uniaxial tensile testing.

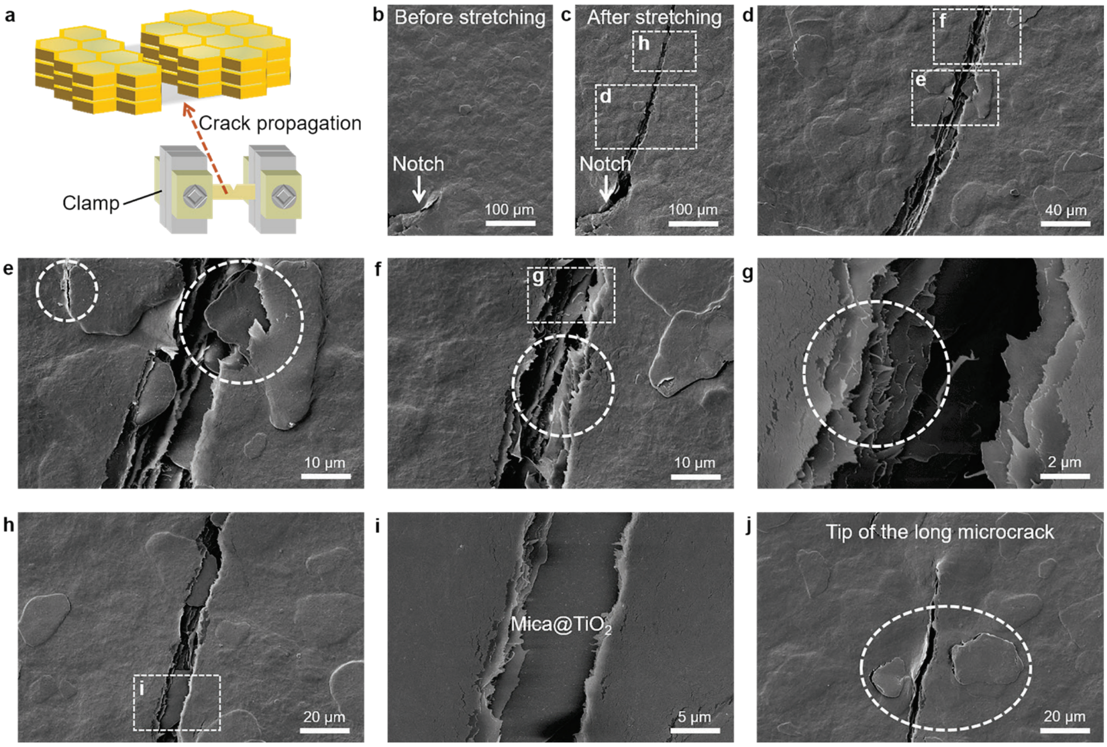

Supplementary Fig. 11. Crack propagation behaviors of AMTA in single-edge notched tensile (SENT) tests. a) Schematic illustration of SENT experiments. **b,c**) FESEM images of notched AMTA before (**b**) and after (**c**) stretching, demonstrating that circuitous crack propagation occurred indeed after stretching the notch. **d**) major crack next to notch. **e**) Pull-out and fracture of Mica@TiO_2 microplatelets. **f,g**) Plastic deformation and curling of dendritic ANFs adhesives at the edge of cracks. **h,i**) Mica@TiO_2 microplatelets span the whole major crack to withstand more stress. **j**) Tip of the long microcrack, showing short cracks were bridged by Mica@TiO_2 on both sides.

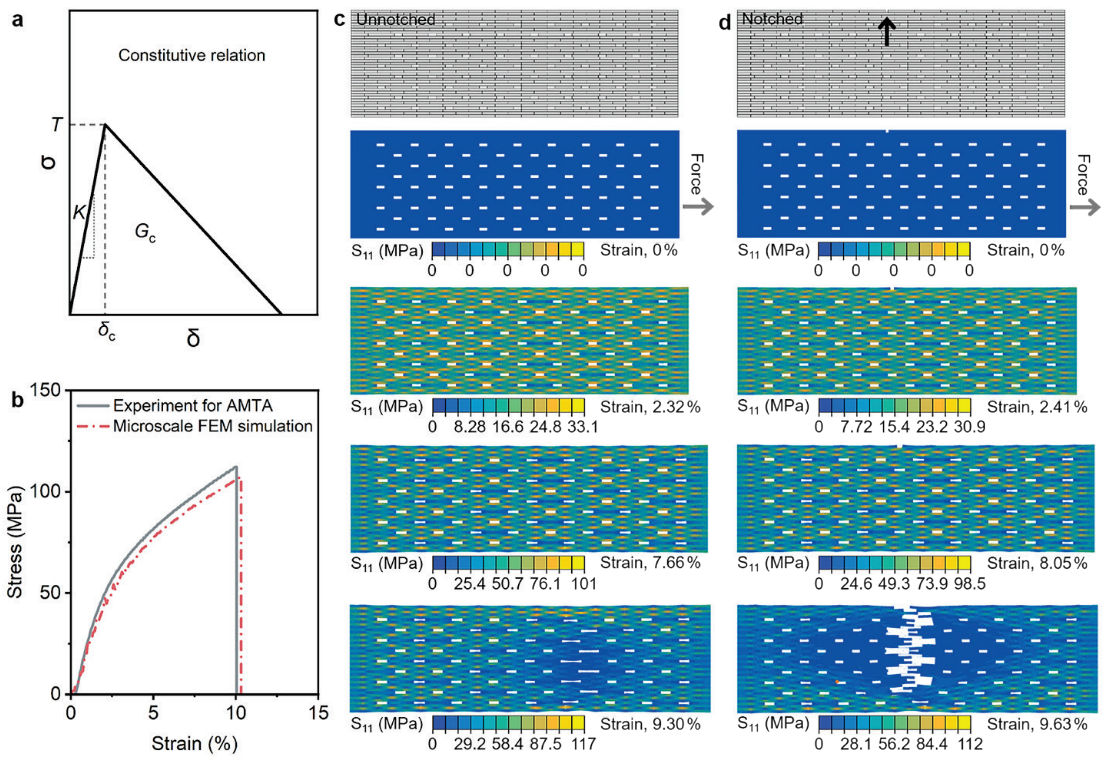

Supplementary Fig. 12. Microscale Finite-Element Analysis of Mechanical Response of AMTA. a) Bilinear traction-separation response of the CZM[3]. **b**) Tensile stress-strain curves of AMTA with porous lamellar microstructures under uniaxial tension mode from experiments and FEM simulation. **c,d**) Stress nephograms of AMTA with (**d**) and without (**c**) a single edge notch during structural deformation and failure process under uniaxial tension. Specifically, significant tensile deformation of cohesive zones (ANFs) and pores occurred, dissipating a large amount of stress, while external toughening by microcracks deflection and bridging was also observed in the Mica@TiO_2-stacked lamellar microstructures.

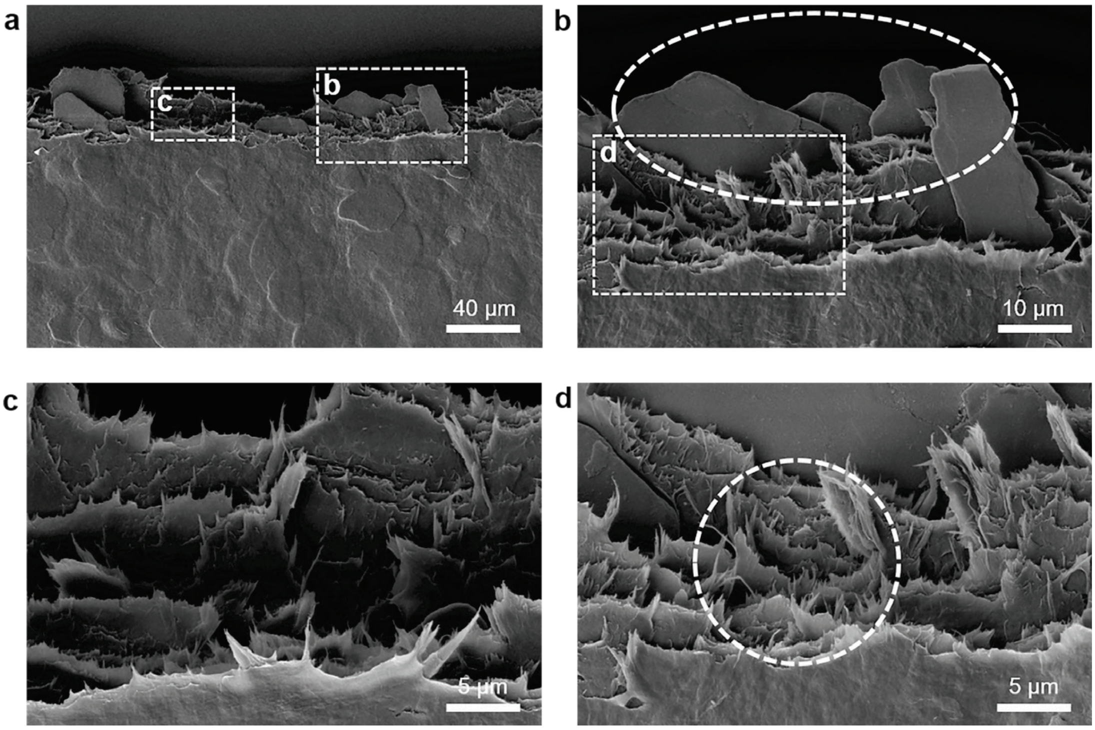

Supplementary Fig. 13. Inclined-view FESEM images of the tensile fracture surface of AMTA. a) Overview of the fracture surface, showing lamellar trapezoidal structure. **b**) Pull-out of Mica@TiO_2 microplatelets and extensive interface delamination toughening[11]. **c,d**) Strong plastic deformation and curling of dendritic ANFs adhesives.

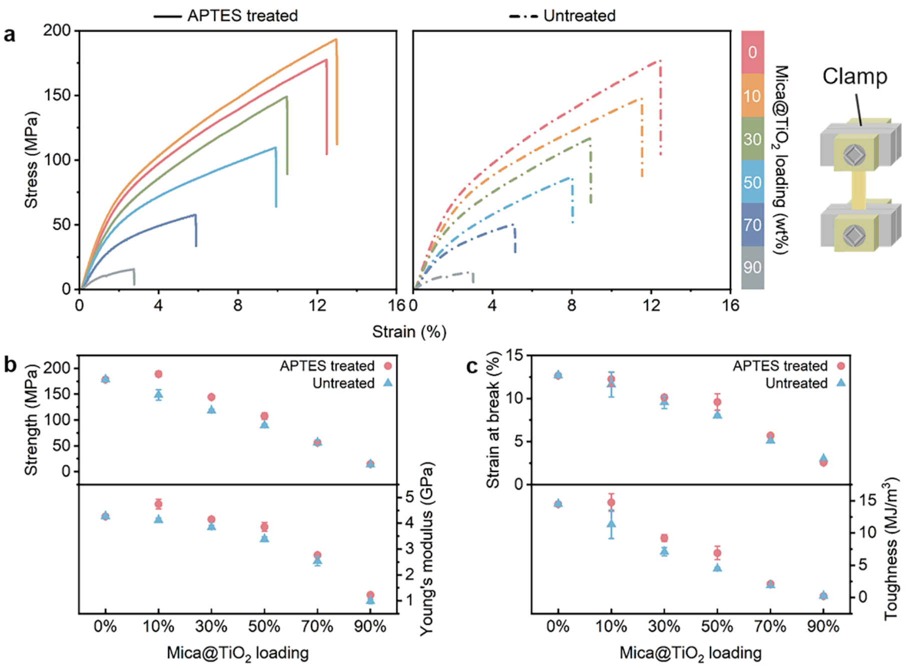

Supplementary Fig. 14. Effect of APTES modification on mechanical properties. **a**) Tensile stress-strain curves of PDRC films with and without APTES treatment. **b,c**) Ultimate strength, maximum young's modulus (**b**) and strain at break, work of toughness (**c**) obtained by uniaxial tensile tests. After being pretreated by APTES, the hydroxyl groups on the surface of Mica@TiO_2 react with the silane groups of APTES (Supplementary Fig. 5), which can facilitate the interfacial interaction between Mica@TiO_2 and ANFs network, resulting in higher mechanical strength, Young's modulus and toughness.

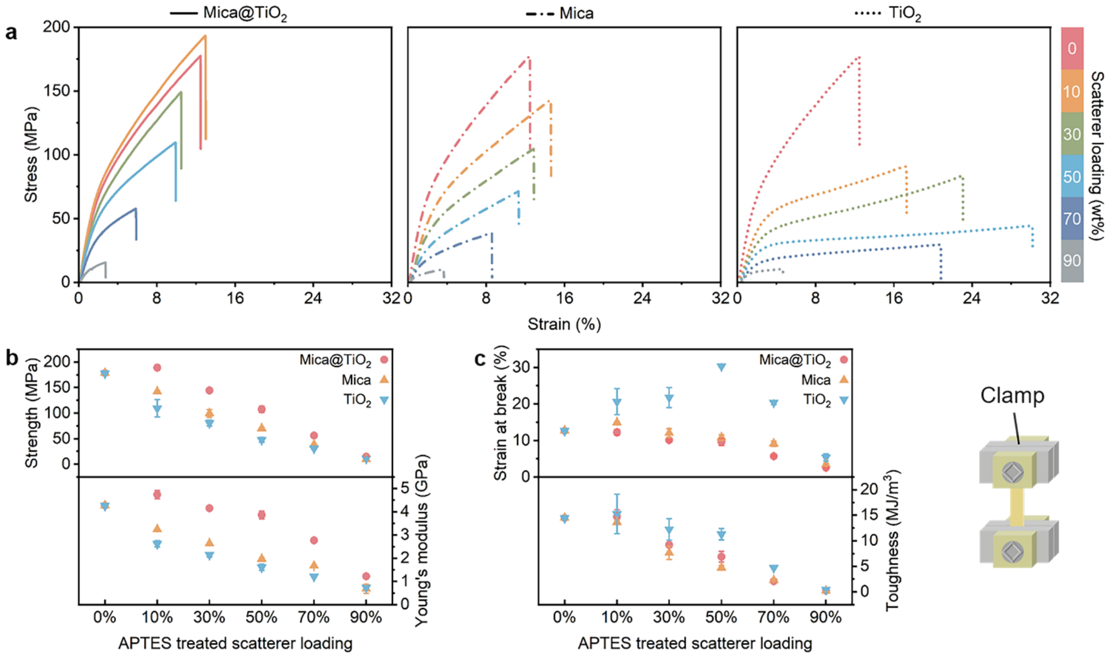

Supplementary Fig. 15. Effect of different dielectric scatterers on mechanical properties. a) Tensile stress-strain curves of PDRC films with different dielectric sactterers. **b,c**) Ultimate strength, maximum young's modulus (**b**) and strain at break, work of toughness (**c**) obtained by uniaxial tensile tests. For different types of dielectric scatterers, such as Mica@TiO_2, Mica and TiO_2, their intrinsic geometry has significant impact on the mechanical performance of these PDRC films. Generally speaking, 2D platelet scatterers with inherent ultra-high aspect-ratio, like Mica@TiO_2 and Mica, exhibit outstanding mechanical properties, especially in strength and modulus. At the same time, the well-organized lamellar microstructure assembled with 2D platelets and ANFs network can further strengthen and toughen these PDRC films[12]. However, for TiO_2 scatterers, the agglomeration phenomenon caused by high specific surface energy of zero-dimensional nanoparticles leads to poor mechanical performance[13] and weak light scattering effect.

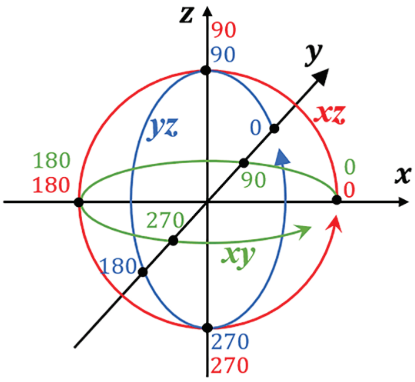

Supplementary Fig. 16. Specified datum-plane in scattering phase function simulated by FDTD, which determines the scattering direction, i.e. forward or backward[14].

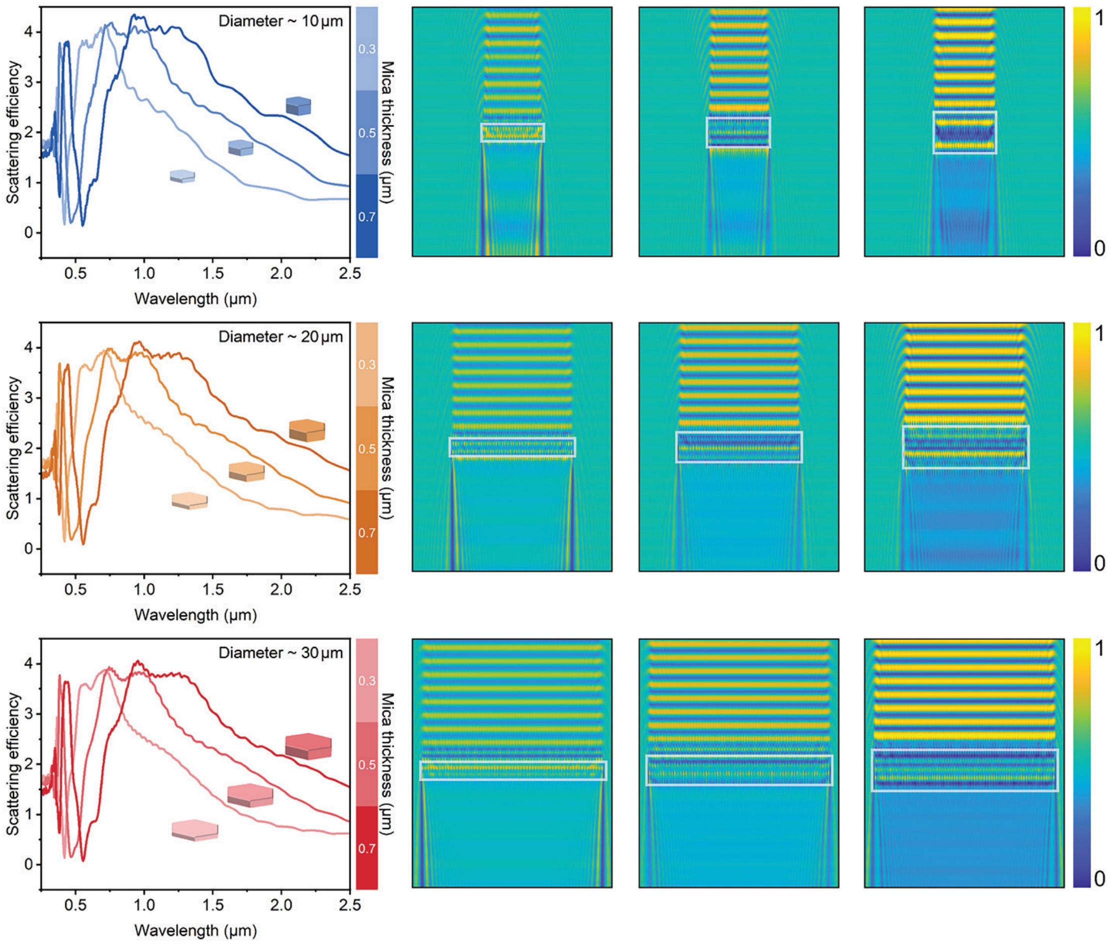

Supplementary Fig. 17. Influence of the diameter and thickness of Mica layer on scattering efficiency and electric field distribution simulated by FDTD, note that the thickness of TiO_2 layer is 50 nm (not shown in the figures for brevity). The left is the scattering efficiency, and the right is the corresponding electric field distributions. It can be observed that the scattering efficiency curves is mainly affected by the thickness of Mica layer and shift to longer wavelength, while the diameter has little influence. Similar to the scattering efficiency, the intensity of the electric field distributions at 0.5 μm wavelength are also determined by the thickness of Mica layer, presenting obvious backward scattering. Therefore, the thickness of Mica layer is crucial to the scattering behaviors of Mica@TiO_2.

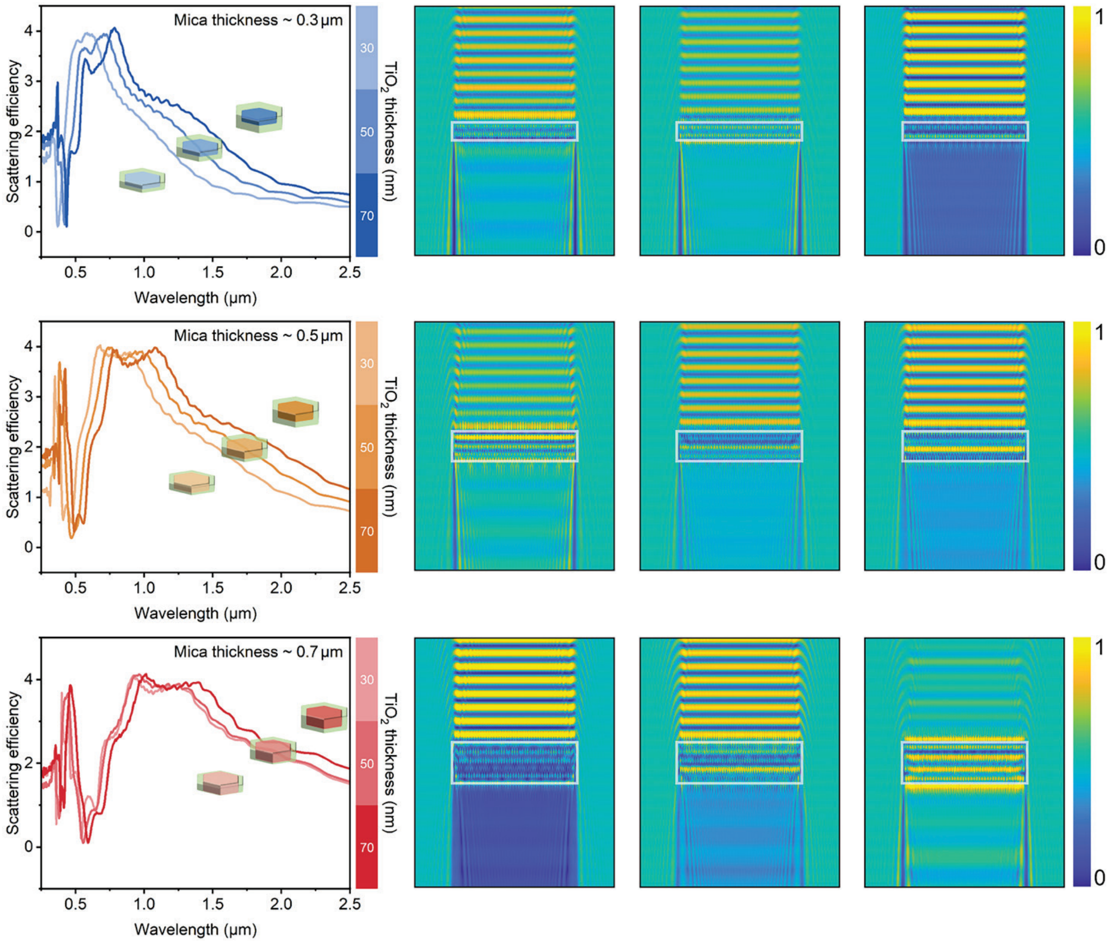

Supplementary Fig. 18. Influence of the thickness of Mica layer and TiO_2 layer on scattering efficiency and electric field distribution simulated by FDTD, note that the diameter is 20 μm. The left is the scattering efficiency, and the right is the corresponding electric field distributions. When the thickness of Mica layer is small (0.3 μm), the overall scattering efficiency of Mica@TiO_2 is greatly affected by the thickness of TiO_2 layer, presenting high values at VIS-NIR band, where the solar irradiation is strongest. Moreover, the thickness of both Mica and TiO_2 layer has a decisive effect on the electric field distributions of Mica@TiO_2 at 0.5 μm wavelength despite the lack of obvious regularities. In general, the combination of high scattering efficiency and effective backward scattering has a great contribution to the final reflectivity of PDRC films based on Mica@TiO_2.

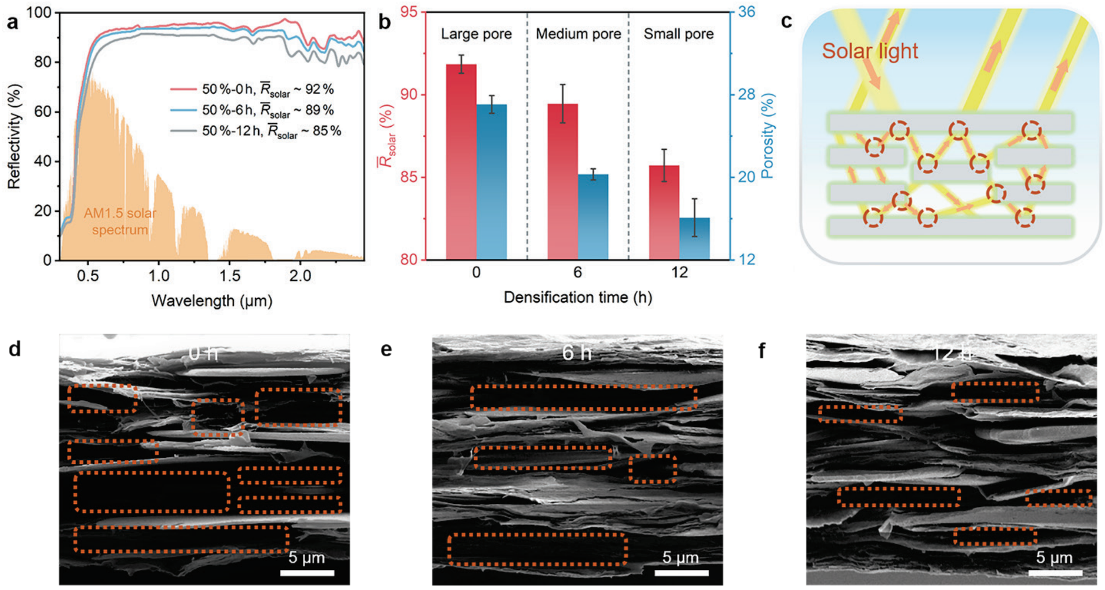

Supplementary Fig. 19. Effect of interlaminar micropores of AMTA on solar reflectivity. a) Solar reflectivity spectra of AMTA with the same thickness (25 μm) but different lamellar porosity caused by the regulation of densification time (20 MPa, 25 °C), AM 1.5 global solar spectrum was shaded as reference. **b**) Calculated $\overline{R}_{solar}$ and porosity of AMTA with different pore size distributions (large, medium and small) obtained by the regulation of densification time. **c**) Schematic illustration of multiple scattering behaviors of AMTA caused by the interlaminar micropores. **d-f**) Section-view FESEM images of AMTA with different pore size distributions obtained by the regulation of densification time.

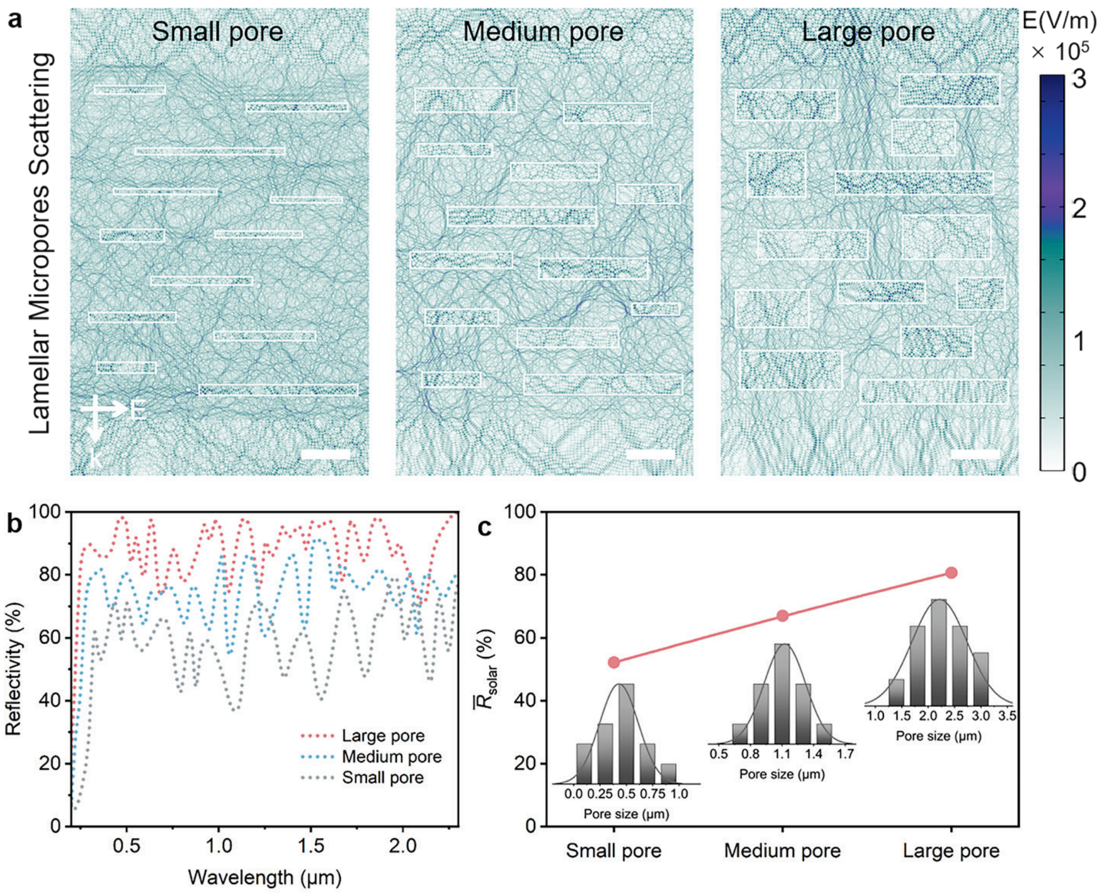

Supplementary Fig. 20. Verification of the interlaminar micropores on solar reflectivity for AMTA by FEM simulations. a-c) Simulated section-view electric field norm distributions (**a**, scale bar, 3 μm.), solar reflectivity spectra (**b**) and calculated $\overline{R}_{solar}$ (**c**, inset is statistical pore size distributions for the small, medium and large pore) of AMTA with different pore size distributions. FEM simulations are consistent with the experimental results in Supplementary Fig. 19, indicating the positive effects of interlaminar micropores on solar reflectivity of AMTA.

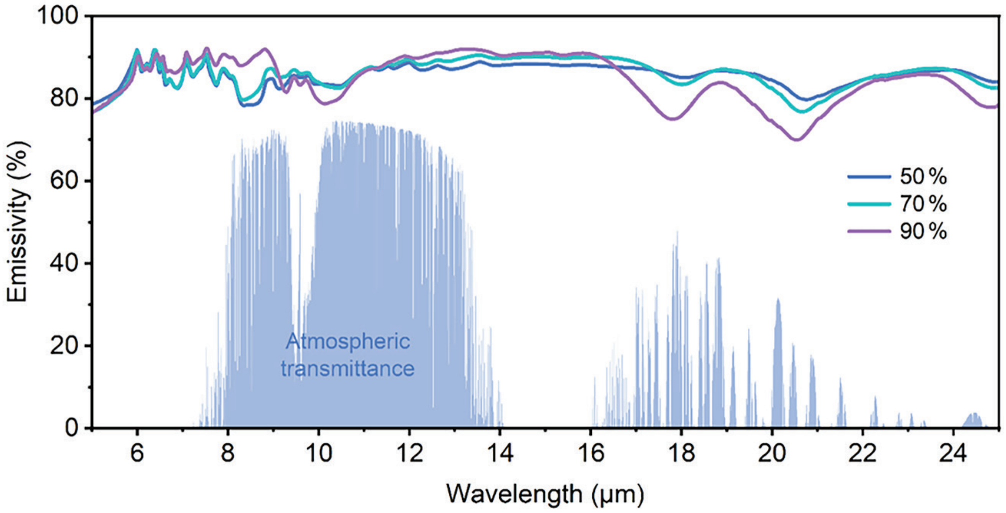

Supplementary Fig. 21. Emissivity spectra of PDRC films with 50, 70 and 90 wt% Mica@TiO_2 loading, showing acceptable $\bar{\varepsilon}_{LWIR}$ (> 85%) for radiative cooling, realistic atmospheric window was shaded as reference.

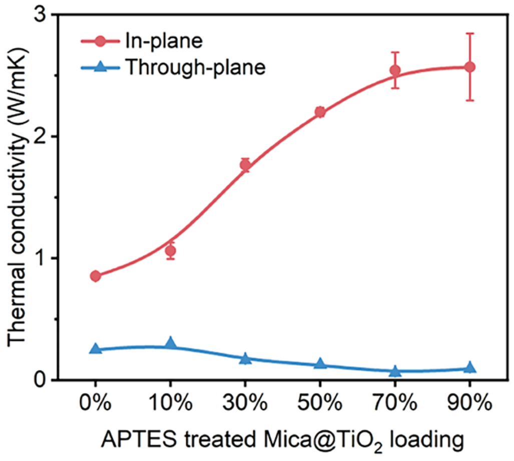

Supplementary Fig. 22. Dependence of in-plane and through-plane thermal conductivities of PDRC films on Mica@TiO_2 loading. When considering above-ambient radiative cooling applications with a large amount of heat generation, high thermal conductivity (> 2 W/mK) of AMTA can assist radiative cooling by means of heat conduction to achieve more efficient cooling, thus avoiding serious security issues.

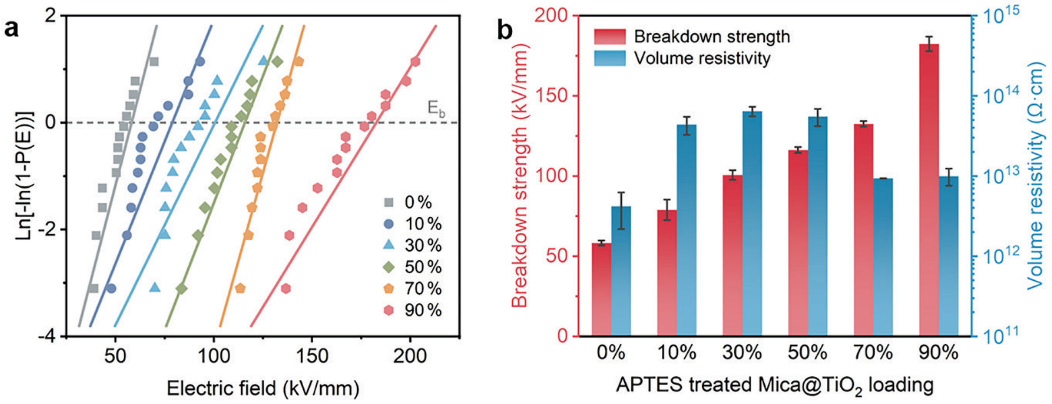

Supplementary Fig. 23. Electrical insulating performance of PDRC films. a) Weibull distribution of examined breakdown strength from PDRC films with different Mica@TiO_2 loading. **b**) Dependence of breakdown strength and volume resistivity of PDRC films on Mica@TiO_2 loading.

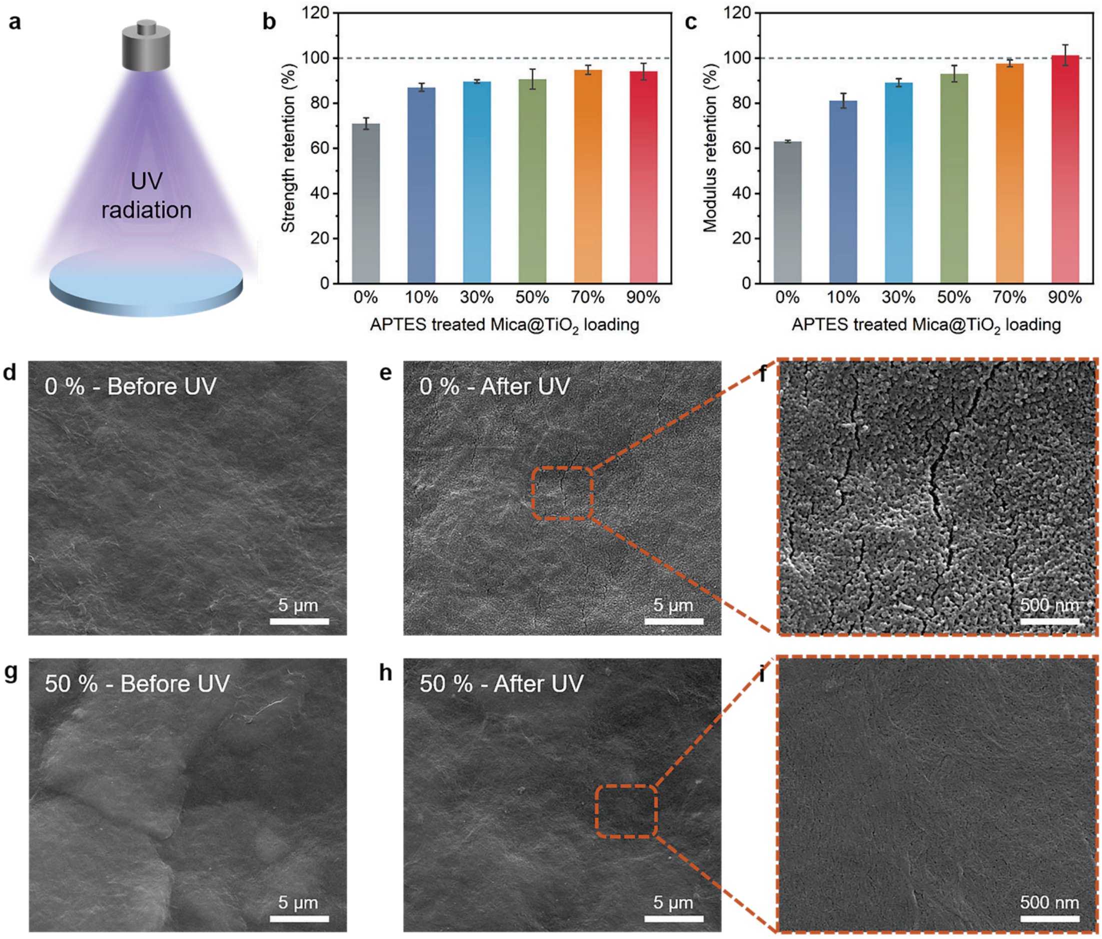

Supplementary Fig. 24. Stability of mechanical properties for PDRC films to UV radiation. a) Schematic illustration of UV radiation tests (iodine lamp with 365 nm maximum intensity, UV irradiance of 10.0 ± 0.5 W/m^2 and 48 h). **b,c**) Retention of strength (**b**) and young's modulus (**c**) for PDRC films with different Mica@TiO_2 loading after UV radiation. **d-i**) Top-view FESEM images with different magnifications of pure ANFs (**d-f**) and AMTA (with 50wt% Mica@TiO_2 loading, **g-i**) before and after UV radiation, respectively. Surfaces of pure ANFs became quite rough, accompanied by a large number of microcracks. In contrast, surfaces of AMTA did not display the above phenomenon, demonstrating its UV stability while working outdoors.

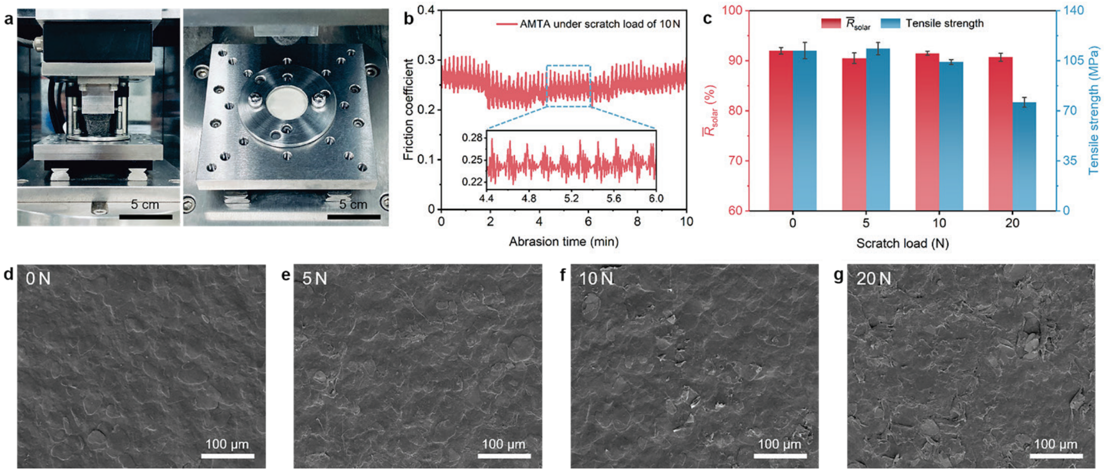

Supplementary Fig. 25. Scratch resistance of AMTA. a) Front and top views of cyclic scratch experiments with a steel wool. **b**) The friction coefficient (~0.25) of AMTA under scratch load of 10 N for 1000 cycles, which is comparable to that of glass and lower than that of most common polymers[15], demonstrating the excellent scratch resistance of AMTA. **c**) The almost unchanged $\overline{R}_{solar}$ and tensile strength of AMTA after bearing 0, 5, 10 and 20 N scratch load for 1000 cycles. **d-g**) FESEM images of AMTA after severe scratch testing, exhibiting no fatal marks on surface even under 10 N scratch load.

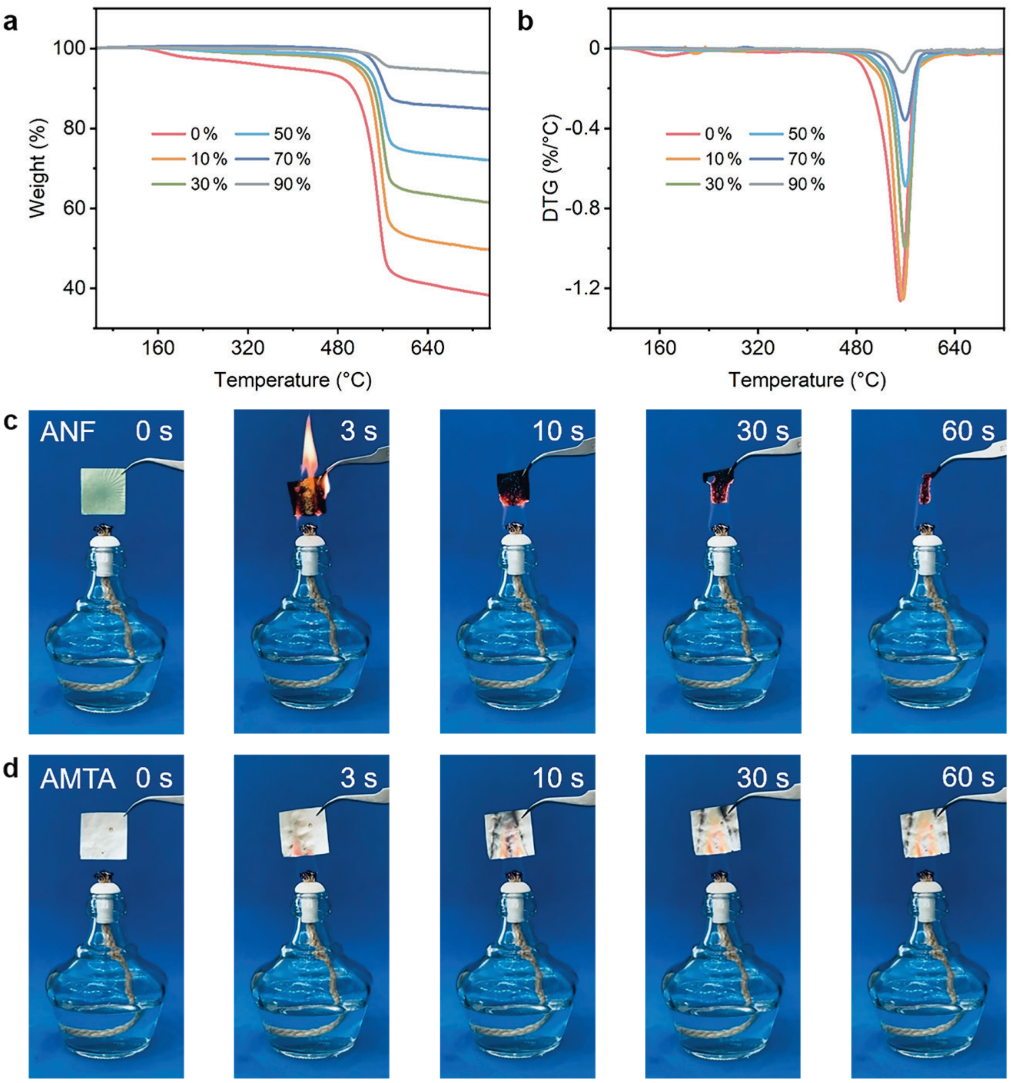

Supplementary Fig. 26. Thermal stability and flame retardancy of AMTA. a,b) TGA (**a**) and derivative thermogravimetry (DTG, **b**) of PDRC films with different Mica@TiO_2 loading. **c,d**) Photographs of pure ANFs (**c**) and AMTA (**d**) in vertical combustion tests, respectively. Compared with pure ANFs, AMTA exhibits excellent flame-retardant and self-extinguished property, demonstrating its safety of fire prevention while working outdoors.

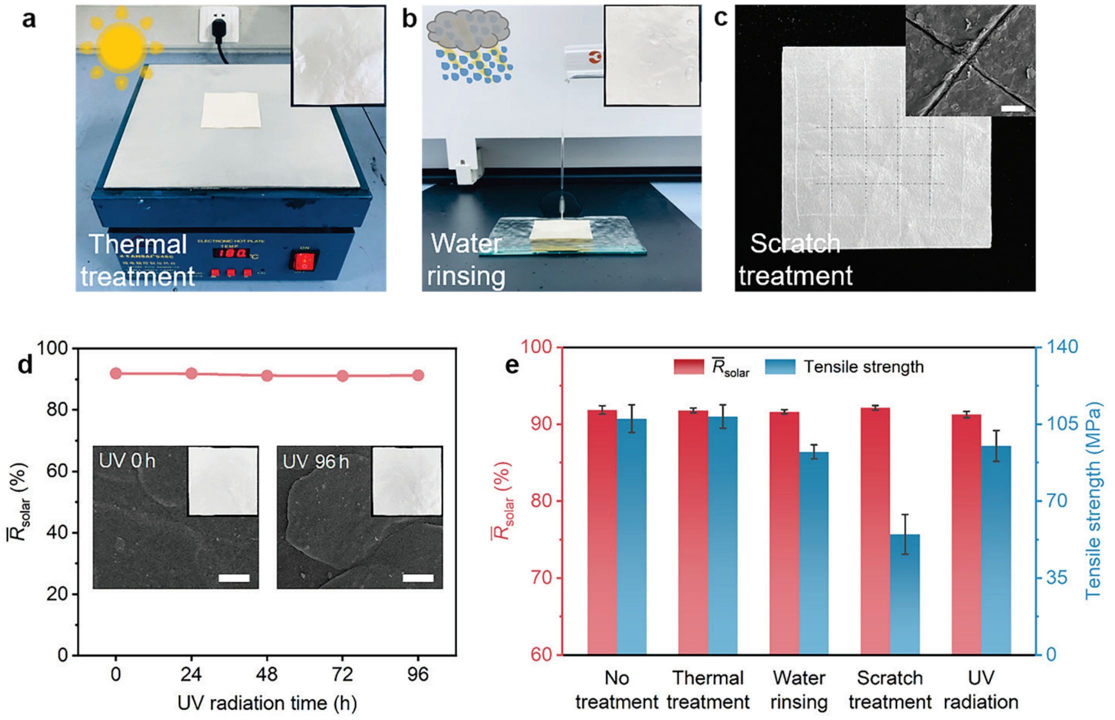

Supplementary Fig. 27. Environmental durability of mechanical and optical properties for AMTA. a-c) Photographs of thermal treatment (**a**, 180 °C for 8 h, inset is a photograph of treated AMTA), water rinsing (**b**, 6 m/s water jet for 8 h, inset is a photograph of treated AMTA) and scratch treatment (**c**, inset is a FESEM image of scratches, scale bar, 80 μm) for AMTA. **d**) Relationship between $\overline{R}_{solar}$ and UV radiation time (insets are photographs and corresponding FESEM images of AMTA before and after 96 h UV radiation, scale bar, 2 μm), showing excellent stability of optical properties to UV radiation. **e**) The almost unchanged $\overline{R}_{solar}$ and tensile strength of AMTA after various extreme weathering treatments.

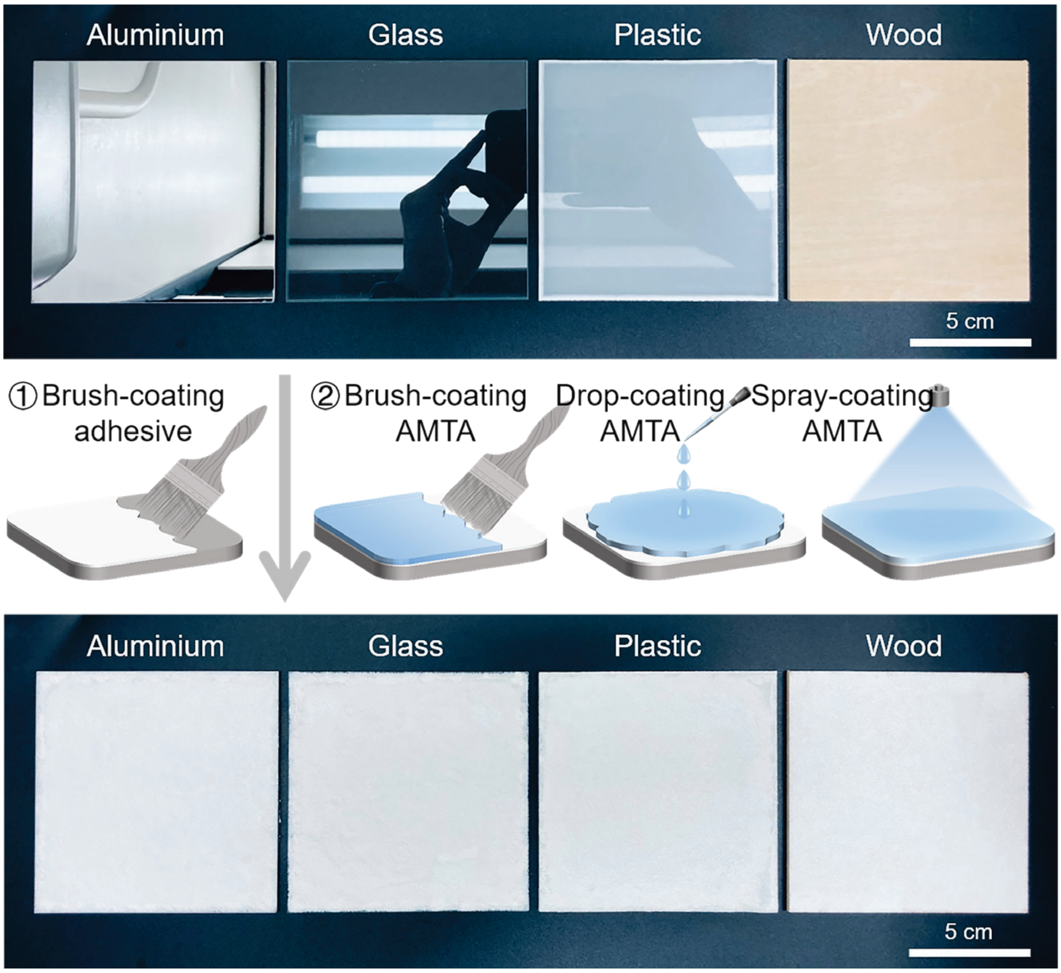

Supplementary Fig. 28. Assembly of AMTA on different substrates. To enhance the adhesion of AMTA to the substrate, a layer of adhesive was first brush-coating to the substrate such as aluminium, glass, plastic, wood, etc. Subsequently, AMTA suspension with different viscosity could be assembled on the substrate by brush-coating, drop-coating or spray-coating according to the construction needs.

Supplementary Table 1. Comparison of comprehensive performance of radiative coolers.

Materials	Thickness (µm)	Strength (MPa)	Reflectivity	Emissivity	Year
PVDF/SiO_2-PTFE	160	2.84	0.8	0.9	2022[16]
PDMS/*h*-BN	1000	3	0.96	0.89	2022[17]
PLA/TiO_2-PTFE	550	44	0.924	0.945	2021[18]
CNC-porous EC	101	18.5	0.951	0.9	2022[19]
PA/PVDF/PE	70	19.8	0.9022	0.75	2020[20]
Porous SEBS/AgNWs	100	1.5	0.92	0.85	2019[21]
Porous P(VDF-HFP)	350	6.5	0.96	0.97	2018[22]
Porous plastics	> 1000	38	0.96	0.9	2021[23]
PVDF/Si_3N_4-PE/PDA	105	57	0.9194	0.8731	2020[24]
Polyester-cotton/$CaCl_2$	314	61	0.9	0.83	2021[25]
**This work (50 wt%)**	**25**	**107.5**	**0.92**	**0.87**	**2023**
**This work (70 wt%)**	**23**	**56.0**	**0.93**	**0.87**	**2023**
**This work (90 wt%)**	**19**	**14.7**	**0.95**	**0.88**	**2023**

Supplementary References

1. Nussenzveig, H.M. & Wiscombe, W.J. Efficiency factors in Mie scattering. *Phys. Rev. Lett.* **45**, 1490-1494 (1980).
2. Hui, C.Y., Ruina, A., Long, R. & Jagota, A. Cohesive zone models and fracture. *J. Adhes.* **87**, 1-52 (2011).
3. Park, K. & Paulino, G.H. Cohesive zone models: A critical review of traction-separation relationships across fracture surfaces. *Appl. Mech. Rev.* **64**(2013).
4. Chen, K., *et al.* Graphene oxide bulk material reinforced by heterophase platelets with multiscale interface crosslinking. *Nat. Mater.* **21**, 1121-1129 (2022).
5. Chen, S.-M., *et al.* Superior biomimetic nacreous bulk nanocomposites by a multiscale soft-rigid dual-network interfacial design strategy. *Matter* **1**, 412-427 (2019).
6. Ji, G., Ouyang, Z. & Li, G. On the interfacial constitutive laws of mixed mode fracture with various adhesive thicknesses. *Mech. Mater.* **47**, 24-32 (2012).
7. Yan, J., *et al.* Understanding the effect of surface/bulk defects on the photocatalytic activity of TiO2: Anatase versus rutile. *Phys. Chem. Chem. Phys.* **15**, 10978-10988 (2013).
8. Zhu, J., *et al.* Strong and stiff aramid nanofiber/carbon nanotube nanocomposites. *ACS Nano* **9**, 2489-2501 (2015).
9. Sun, W.-B., *et al.* Nacre-inspired bacterial cellulose/Mica nanopaper with excellent mechanical and electrical insulating properties by biosynthesis. *Adv. Mater.* **35**, 2300241 (2023).
10. Wan, S., *et al.* High-strength scalable MXene films through bridging-induced densification. *Science* **374**, 96-99 (2021).
11. Gao, H.-L., *et al.* Mass production of bulk artificial nacre with excellent mechanical properties. *Nat. Commun.* **8**, 287 (2017).
12. Li, J., Liu, X., Feng, Y. & Yin, J. Recent progress in polymer/two-dimensional nanosheets composites with novel performances. *Prog. Polym. Sci.* **126**, 101505 (2022).
13. Zare, Y. Study of nanoparticles aggregation/agglomeration in polymer particulate nanocomposites by mechanical properties. *Composites Part A* **84**, 158-164 (2016).

14. Mie scattering (fdtd). in *Ansys Optics* (Ansys Optics, https://optics.ansys.com/hc/en-us/articles/360042703433).
15. Ludema, K.C. & Tabor, D. The friction and visco-elastic properties of polymeric solids. *Wear* **9**, 329-348 (1966).
16. Yang, C.*, et al.* Scalable fabrication of PVDF/SiO2-PTFE fiber membrane for effective daytime radiative cooling. *Mater. Lett.* **320**, 132372 (2022).
17. Li, P.*, et al.* Thermo-optically designed scalable photonic films with high thermal conductivity for subambient and above-ambient radiative cooling. *Adv. Funct. Mater.* **32**, 2109542 (2022).
18. Zeng, S.*, et al.* Hierarchical-morphology metafabric for scalable passive daytime radiative cooling. *Science* **373**, 692-696 (2021).
19. Zhu, W.*, et al.* Structurally colored radiative cooling cellulosic films. *Adv. Sci.* **9**, 2202061 (2022).
20. Song, Y.-N., Li, Y., Yan, D.-X., Lei, J. & Li, Z.-M. Novel passive cooling composite textile for both outdoor and indoor personal thermal management. *Composites Part A* **130**, 105738 (2020).
21. Xu, Y.*, et al.* Multiscale porous elastomer substrates for multifunctional on-skin electronics with passive-cooling capabilities. *Proc. Natl. Acad. Sci. U.S.A.* **117**, 205-213 (2020).
22. Mandal, J.*, et al.* Hierarchically porous polymer coatings for highly efficient passive daytime radiative cooling. *Science* **362**, 315-319 (2018).
23. Gao, W., Lei, Z., Wu, K. & Chen, Y. Reconfigurable and renewable nano-micro-structured plastics for radiative cooling. *Adv. Funct. Mater.* **31**, 2100535 (2021).
24. Song, Y.-N., Lei, M.-Q., Deng, L.-F., Lei, J. & Li, Z.-M. Hybrid metamaterial textiles for passive personal cooling indoors and outdoors. *ACS Appl. Polym. Mater.* **2**, 4379-4386 (2020).
25. Sun, Y.*, et al.* Preparation of passive daytime cooling fabric with the synergistic effect of radiative cooling and evaporative cooling. *Adv. Mater. Technol.* **7**, 2100803 (2022).
