# Supplementary material for: Thin lamellar films with enhanced mechanical properties for durable radiative cooling
Source: Nat Commun. 2023 Oct 2;14:6129. doi: 10.1038/s41467-023-41797-3 (PMC10545832; doi:10.1038/s41467-023-41797-3)
Supplement: Supplementary file 3 — Description of Additional Supplementary Files [file 41467_2023_41797_MOESM3_ESM.pdf]

Description of Additional Supplementary Files

File Name: Supplementary Movie 1
Description: Scattering behaviors of Mica@TiO_2 simulated by FDTD.

File Name: Supplementary Movie 2
Description: Scattering behaviors of Mica simulated by FDTD.

File Name: Supplementary Movie 3
Description: Scattering behaviors of TiO_2 simulated by FDTD.
