# Supplementary material for: Thin lamellar films with enhanced mechanical properties for durable radiative cooling
Source: Nat Commun. 2023 Oct 2;14:6129. doi: 10.1038/s41467-023-41797-3 (PMC10545832; doi:10.1038/s41467-023-41797-3)
Supplement: Supplementary file 7 — Reporting Summary [file 41467_2023_41797_MOESM7_ESM.pdf]

nature portfolio

Corresponding author(s): Qiang Fu; Hua Deng

Last updated by author(s): Aug 25, 2023

Reporting Summary

Nature Portfolio wishes to improve the reproducibility of the work that we publish. This form provides structure for consistency and transparency in reporting. For further information on Nature Portfolio policies, see our Editorial Policies and the Editorial Policy Checklist.

Statistics

For all statistical analyses, confirm that the following items are present in the figure legend, table legend, main text, or Methods section.

n/a	Confirmed	
☒	☐	The exact sample size (*n*) for each experimental group/condition, given as a discrete number and unit of measurement
☒	☐	A statement on whether measurements were taken from distinct samples or whether the same sample was measured repeatedly
☒	☐	The statistical test(s) used AND whether they are one- or two-sided<br>*Only common tests should be described solely by name; describe more complex techniques in the Methods section.*
☒	☐	A description of all covariates tested
☒	☐	A description of any assumptions or corrections, such as tests of normality and adjustment for multiple comparisons
☒	☐	A full description of the statistical parameters including central tendency (e.g. means) or other basic estimates (e.g. regression coefficient) AND variation (e.g. standard deviation) or associated estimates of uncertainty (e.g. confidence intervals)
☒	☐	For null hypothesis testing, the test statistic (e.g. *F*, *t*, *r*) with confidence intervals, effect sizes, degrees of freedom and *P* value noted<br>*Give P values as exact values whenever suitable.*
☒	☐	For Bayesian analysis, information on the choice of priors and Markov chain Monte Carlo settings
☒	☐	For hierarchical and complex designs, identification of the appropriate level for tests and full reporting of outcomes
☒	☐	Estimates of effect sizes (e.g. Cohen's *d*, Pearson's *r*), indicating how they were calculated

Our web collection on statistics for biologists contains articles on many of the points above.

Software and code

Policy information about availability of computer code

Data collection	The simulations covered in the manuscript were performed using commercial software as follows: FDTD Solutions 8.19 software by Lumerical Co. Ltd; COMSOL Multiphysics 5.6; ABAQUS/CAE (v2016; Dassault Systemes Simulia, Johnston, RI, USA).
Data analysis	The data analysis covered in the manuscript was performed using commercial software as follow: OriginPro 2021 (9.8.0.200).

For manuscripts utilizing custom algorithms or software that are central to the research but not yet described in published literature, software must be made available to editors and reviewers. We strongly encourage code deposition in a community repository (e.g. GitHub). See the Nature Portfolio guidelines for submitting code & software for further information.

Data

Policy information about availability of data

All manuscripts must include a data availability statement. This statement should provide the following information, where applicable:

- Accession codes, unique identifiers, or web links for publicly available datasets
- A description of any restrictions on data availability
- For clinical datasets or third party data, please ensure that the statement adheres to our policy

The data that support the findings of this study are available from the corresponding authors upon request.

Research involving human participants, their data, or biological material

Policy information about studies with human participants or human data. See also policy information about sex, gender (identity/presentation), and sexual orientation and race, ethnicity and racism.

Reporting on sex and gender	This finding deals only with the field of synthetic materials (not biological material) and not with sex or gender. So there is a lack of sex- and gender-based analysis.
Reporting on race, ethnicity, or other socially relevant groupings	This finding deals only with the field of synthetic materials (not biological material) and not with race, ethnicity, or other socially relevant groupings. So there is a lack of reporting on race, ethnicity, or other socially relevant groupings.
Population characteristics	This finding deals only with the field of synthetic materials (not biological material) and not with human research participants. So there is a lack of population characteristics.
Recruitment	This finding deals only with the field of synthetic materials (not biological material) and not with human research participants. So there is a lack of recruitment.
Ethics oversight	This finding deals only with the field of synthetic materials (not biological material) and not with human participants or biological material. So there is a lack of ethics oversight.

Note that full information on the approval of the study protocol must also be provided in the manuscript.

Field-specific reporting

Please select the one below that is the best fit for your research. If you are not sure, read the appropriate sections before making your selection.

☐ Life sciences ☐ Behavioural & social sciences ☒ Ecological, evolutionary & environmental sciences

For a reference copy of the document with all sections, see nature.com/documents/nr-reporting-summary-flat.pdf

Ecological, evolutionary & environmental sciences study design

All studies must disclose on these points even when the disclosure is negative.

Study description	A novel passive daytime radiative cooling material was designed and developed, its various properties such as optical, mechanical, and weathering resistance were investigated, and its internal mechanisms were hypothesized through comparative experiments and optical simulations.
Research sample	All samples were prepared independently by us and the raw materials were obtained from commercially available products (described in detail in the "Methods" section of the manuscript), and no biological materials were involved. Subsequent experimental tests and related data were based on these samples (described in detail in the "Methods" section of the manuscript).
Sampling strategy	The samples in this study were derived from synthetically prepared materials and each set of samples was repeated more than 5 times to ensure reliability. No sampling was involved.
Data collection	Based on the sample material we prepare, data is collected through a range of test methods (standard or in-house instruments). Most of the data collection was done by Lianhu Xiong (some data was done by qualified testing organizations).
Timing and spatial scale	The data in this study were derived from various tests and are not necessarily related to each other. Therefore, most of the test data do not need to be sampled at regular intervals, except for the UV aging test, which is sampled at 24-hour intervals.
Data exclusions	No data were artificially excluded from the analyses.
Reproducibility	All samples were prepared and tested at least 5 times with sufficient reproducibility (the experimental methods are described in detail in the "Methods" section of the manuscript).
Randomization	This finding deals only with the field of synthetic materials (not biological material) and not with organisms or participants. So there is a lack of random sampling.
Blinding	This finding deals only with the field of synthetic materials (not biological material) and not with organisms or participants. So there is a lack of blinding.

Did the study involve field work? ☐ Yes ☒ No

Reporting for specific materials, systems and methods

We require information from authors about some types of materials, experimental systems and methods used in many studies. Here, indicate whether each material, system or method listed is relevant to your study. If you are not sure if a list item applies to your research, read the appropriate section before selecting a response.

Materials & experimental systems

n/a	Involved in the study
☒	☐ Antibodies
☒	☐ Eukaryotic cell lines
☒	☐ Palaeontology and archaeology
☒	☐ Animals and other organisms
☒	☐ Clinical data
☒	☐ Dual use research of concern
☒	☐ Plants

Methods

n/a	Involved in the study
☒	☐ ChIP-seq
☒	☐ Flow cytometry
☒	☐ MRI-based neuroimaging
